# Pediatric Multiple Sclerosis and Cognition: A Review of Clinical, Neuropsychologic, and Neuroradiologic Features

**DOI:** 10.1155/2017/1463570

**Published:** 2017-12-25

**Authors:** Ozgul Ekmekci

**Affiliations:** Department of Neurology, Faculty of Medicine, Ege University, Izmir, Turkey

## Abstract

Multiple sclerosis (MS) is an inflammatory demyelinating and neurodegenerative disease. Although cognitive impairment has been well established in adult patients with MS, its occurrence in patients with pediatric-onset MS has recently been reported. In this review, I discuss the main features of cognitive impairment in pediatric MS as determined by long-term follow-up studies, neuropsychiatric test batteries, and the results of neuroradiological imaging studies that investigated the pathogenesis of pediatric MS. The most commonly affected cognitive domains in adults are attention, processing speed, and visuomotor skills; language and intelligence are also affected in pediatric MS. A young age at disease onset is the strongest risk factor for these impairments, which may be due to the effect of inflammatory demyelination and neurodegeneration on the developing central nervous system and neural networks in children. Cognitive impairment has long-term effects on patients' academic life and the quality of their social life. Therefore, all patients with pediatric MS should be screened and monitored for cognitive impairment. This review also highlights the need for neuropsychological test batteries that assess different cognitive domains in children and adolescents with multiple sclerosis and for cognitive rehabilitation programs to improve the quality of their academic and social life.

## 1. Introduction

Multiple sclerosis (MS) is an autoimmune-mediated neurodegenerative and demyelinating disorder of the central nervous system primarily affecting young adults. Approximately 2–5% of MS patients have onset before age 18 [1, 2]. The International Pediatric Multiple Sclerosis Study Group defined diagnostic criteria for pediatric MS [3]. MS can cause both physical and cognitive disability. The occurrence of cognitive impairment is well established in adult patients with MS, occurring in approximately 45–65% of these patients [4]. The cognitive domains that are most commonly affected in adult MS patients are complex attention, information processing speed, episodic memory, visuospatial abilities, and executive functions [5]. Cognitive impairment in pediatric MS patients has been described with different studies reporting cognitive impairment in 30–50% of cases [5–8]. Cognitive impairment in pediatric multiple sclerosis differs from that in adults in terms of the cognitive domains involved. Factors such as age, maturation of the central nervous system, and cognitive reserve have different effects on cognition in childhood. The maturation status of the central nervous system at the time of MS onset is a long-term prognostic factor, and it is crucial to develop appropriate neuropsychological test batteries that assess different cognitive domains in children and adolescents with MS. Moreover, cognitive rehabilitation to improve the quality of academic and social life in pediatric MS patients has not yet been well studied. In this review, I discuss the main features of cognitive impairment in pediatric MS, long-term follow-up findings, neuropsychiatric test batteries, and the result of neuroradiological imaging studies that investigated the pathogenesis of pediatric MS.

## 2. The Main Features of Cognitive Impairment in Pediatric MS Patients

Banwell and Anderson were the first to use a neuropsychological battery to systematically investigate a cohort of patients with pediatric MS. They assessed cognitive functions in 10 children with MS and identified deficits in general cognition, language, visuomotor integration, and verbal and visual memory. Children with longer disease duration demonstrated deficits in executive functions, information processing speed, and working memory, suggesting that children with longer disease duration and a younger age at onset are at higher risk for cognitive impairment [6].

MacAllister et al. evaluated 37 children and adolescents with MS and reported significant cognitive impairment in 35% of these patients. The most common impairment was in complex attention. They also found receptive language problems and poor naming in many patients, whereas verbal fluency was unaffected. Immediate verbal memory was impaired in only one patient; however, delayed recall was impaired in seven patients. The researchers suggested that, in these patients, the effect of MS on attention and memory was similar to that in adults with MS, but visuospatial functions were less affected. They found a strong relationship between cognitive impairment and Expanded Disability Status Scale (EDSS) score, total number of relapses, and disease duration [7].

In a multicenter study, Amato and colleagues assessed the frequency, pattern, and clinical correlates of cognitive impairment in childhood and adolescent MS cases and compared these characteristics with those in healthy controls. The researchers used a neuropsychological battery to assess intelligence quotient (IQ), memory, attention/concentration, executive functions, and language. They found significant cognitive impairment in 31% of the patients with MS, and 53% of the cases failed at least two tests. Both verbal and performance IQ scores were lower in MS patients than in healthy controls. The most frequently affected cognitive domains were memory, particularly visuospatial memory, complex attention, verbal comprehension, and executive functions. In this study, EDSS and number of relapses were not adequate descriptors of cognitive condition; cognitive impairments were found also in patients with low disability levels. In this cohort, there was a significant correlation between cognitive impairment and low IQ scores. Low IQ score was also significantly associated with younger age at disease onset [8].

In a study of the magnetic resonance imaging (MRI) correlates of cognitive impairment in pediatric MS patients, Till et al. found impairments in attention and processing speed, visuomotor integration, and expressive language. They observed cognitive impairment in 29.4% of the MS patients. Full-scale IQ and verbal IQ were significantly reduced in children with MS and a higher IQ was associated with a shorter disease duration and an older age at disease onset. Moreover, a higher full-scale IQ was correlated with a lower EDSS score, and patients with cognitive impairment tended to be male. Finally, cognitive impairment was associated with a longer disease duration and a younger age at onset [9].

In a large multicenter study, Julian et al. examined cognitive function in 231 pediatric patients with MS (*n* = 187) or clinically isolated syndrome (*n* = 44). The cognitive impairment was noted in 35% of patients with MS and 18% of patients with clinically isolated syndrome. The cognitive areas that were most frequently affected were fine motor coordination (54%), visuomotor integration (50%), and information processing speed (35%). EDSS score was the only variable that was significantly and independently associated with reduced cognitive function [10].

The methods and results of these cross-sectional studies are shown in Table 1. Although these studies used different neuropsychological tests and different definitions of cognitive impairment, they found similar rates of cognitive impairment. The rate of cognitive impairment in pediatric MS ranged from 29.4% to 35%. Full-scale IQ and verbal and performance IQ were lower in children with MS. Low IQ scores were associated with higher EDSS score and young age at disease onset [8, 9]. The relationship of cognitive impairment with young age at onset and disease duration was similar in many studies [6–9]. However, the relationship between EDSS score and cognitive impairment varied between these studies. For example, Julian et al. and Banwell and Anderson found that EDSS score was associated with cognitive impairment but other studies did not [6, 10]. These studies suggested that cognitive impairment affected academic and social activities negatively and impaired the quality of life [6–8].

In summary, similar to adult patients, pediatric MS patients experience deficits in attention, information processing speed, memory, executive functions, and visuomotor integration. Additionally, unlike in adults, MS affects language functions and intelligence occurs in particularly young children. The relationship between cognitive dysfunction and disease duration, disability scores, and relapse frequency is unclear. The strongest predictor is a younger age at disease onset. Although the relationship between motor disability and cognitive disability is controversial, cognitive impairment could occur independently from motor disability. Cognitive impairment affects academic and social activities and quality of life. Monitoring of cognitive impairment in pediatric MS patients is important to enable optimal treatment and rehabilitation for prevention of further cognitive disability.

Another factor affecting MS-induced cognitive functions is cognitive reserve. The cognitive reserve theory states that individual differences in how tasks are processed or neural networks are used can allow some people to cope better with brain pathology than others [11]. It has been shown that both heritable (brain reserve) and environmental (greater intellectual enrichment) factors may attenuate the negative effects of MS on cognitive status in adults [12]. The role of cognitive reserve in pediatric MS is unclear. Pastò et al. assessed the potential impact of cognitive reserve on cognition in a cohort of pediatric MS patients. A total of 48 pediatric MS patients and 57 healthy controls were included in this study, and patients were followed up for 4.7 ± 0.4 years. Cognitive reserve was estimated by measuring the IQ at baseline, and cognitive impairment was defined as failure in ⩾3 tests of an extensive neuropsychological battery that included the Selective Reminding Test (SRT) and Selective Reminding Test-Delayed (SRT-D), the 10/36 Spatial Recall Test (SPART), the 10/36 Spatial Recall Test-Delayed, the Symbol Digit Modalities Test (SDMT), the Trail Making tests (TMT-A and TMT-B), a Semantic and Phonemic Verbal Fluency Test, an Oral Denomination Test from the Aachener Aphasia Test, the Token Test, the Indication of Pictures from the Neuropsychological Examination for Aphasia, and Phrase Comprehension Test from the Battery for the Analysis of Aphasic Deficits. The reliable change index (RCI) method was used to assess changes in neuropsychological performance. Cognitive impairment was found in 29.2% of patients with pediatric onset MS at baseline. Deterioration in cognitive performance was detected using the RCI method in 37.6% patients with pediatric onset MS. A higher cognitive reserve predicted stable or improving performance, whereas a lower reserve was associated with deteriorating performance at follow-up [13]. In other studies, on traumatic brain injury, higher cognitive reserve was associated with higher performance and a greater resistance to the development cognitive deficits [13–15]. Thus, measurement of cognitive reserve can be used to identify patients at high risk for cognitive impairment. Alternatively, cognitive reserve can be used to develop cognitive-protective approaches and cognitive rehabilitation programs.

Compared to adults, children are better able to compensate for brain damage because of the greater neural plasticity of the developing brain. Pediatric MS occurs during periods of brain growth, myelination, and neural network maturation; therefore, longitudinal follow-up studies are important to understanding the interactions between progressive brain pathology and maturation, neural plasticity, and compensatory abilities. During 2- and 5-year follow-ups of childhood and adolescent MS cases in their multicenter study, Amato et al. used the same neuropsychological battery as that used at baseline, with alternative versions of the tests to assess memory, attention/concentration, executive functions, and language. At the 2-year follow-up, 70% of patients failed at least three tests, and overall, 75% of the cases were classified as having deteriorating cognitive performance. Failure was more prominent in tests of verbal memory, complex attention, verbal fluency, and receptive language. An individual cognitive change index was also calculated to assess the direction of change in cognition over the follow-up period. At the 2-year follow-up, cognitive deterioration was observed in 75% of the patients, while 25% of the cases were classified as stable or improving. At the 5-year follow-up, the cognitive impairment index decreased in 56% of the patients, improved in 25%, and remained stable in 18.8%. Cognitive deterioration was associated with male sex, younger age at disease onset, and lower educational level. The cognitive domains with the most tendencies toward deterioration were visuospatial learning and expressive language [16, 17].

In a small cohort of patients with pediatric MS, MacAllister et al. assessed cognitive function longitudinally and found deficits in attention, executive functions, and memory. Their findings suggested that neurological impairment early in the course of the disease was predictive of cognitive decline over time. There was no strong association between duration of disease and cognitive decline. The results suggested that, in patients with pediatric MS, cognition might deteriorate over time relative to that of their peers and their baseline performance [18].

In a longitudinal study, Charvet et al. evaluated cognitive function in 62 patients with pediatric MS and five with clinically isolated syndrome. The researchers evaluated cognition at baseline and at follow-up, with a mean follow-up time of 1.64 ± 0.63 years. Cognitive impairment was detected in 37% of patients at baseline and 33% of patients at follow-up. The most commonly impaired cognitive functions included visuomotor integration, information processing speed, and attention. The researchers indicated that cognitive performance might remain stable, or even improve, over a short period of time [19].

In another longitudinal study, Hosseini et al. investigated how the age at disease onset and cognitive reserve may affect cognitive maturation in children and adolescents with pediatric MS. They used two standardized and widely used neuropsychological measures (SDMT and TMT) to assess visual scanning, information processing speed, and working memory. Cognitive reserve capacity was assessed using the patient's baseline full-scale IQ and parental social status. They used growth curve analysis to evaluate cognitive maturation over childhood and adolescence. Their results showed that a younger age at disease onset was associated with decreased performance on neuropsychological measures (SDMT and TMT). A younger age of disease onset was also associated with lower developmental gain on the neuropsychological measures. Cognitive reserve, including baseline full-scale IQ and parental social status, did not protect against cognitive deterioration [20].

Till et al. investigated the extent, pattern, and correlates of change in cognitive functioning over an approximately 15-month interval in 28 patients with pediatric MS and compared their findings with data from 26 healthy controls. The RCI was used to determine individual differences on test scores over time in this prospective longitudinal study. Participants were categorized as showing either “decline” or “improvement” if changes in scores exceeded the RCI on three or more tests. They observed that healthy controls were more likely to show improvement across multiple domains of function than patients with pediatric MS were. When cognitive change was examined on an individual basis, cognitive deterioration was observed in seven of 28 patients with pediatric MS and only one of 26 healthy controls. Clinically significant improvement on three or more of the neuropsychological tests was detected in 69.2% of healthy controls compared with only 17.9% of pediatric MS patients. Patients with pediatric MS showed decline mainly on tests of information processing speed and attention, visuomotor integration, verbal fluency, abstract visual memory, calculation, and spelling. An association was found between disease duration and deterioration in visuomotor integration. The results showed that patients who were classified as having declined cognitive function (*n* : 7) did not differ from those with stable/improved cognitive function (*n* : 21) with regard to age at testing, age at disease onset, disease duration, sex, mood-related symptoms, baseline IQ, and EDSS scores. The researchers reported that patients with pediatric MS failed to demonstrate age-expected improvements in several domains of cognitive performance [21].

In a study investigating long-term outcomes in adult patients with pediatric-onset MS, SDMT scores were lower in patients with pediatric onset than adult onset both in unadjusted analysis and after adjustment for disease duration. The researchers suggested that, in adulthood, pediatric-onset MS may result in greater impairment in processing speed and that further investigation of other cognitive domains was warranted in this group of patients [22].

The methods and results of longitudinal follow-up studies are shown in Table 2. The duration of follow-up in these studies varied between 1 and 5 years. In Amato et al.'s study, most of the patients (75%) showed deteriorating performance between baseline and year 2; however, comparing baseline and 5-year testing, cognitive impairment index deterioration was observed in 56% of the patients, improvement in 25%, and stability in 18.8% [16, 17]. Charvet et al. emphasized that most of the cases had a stable cognitive function in the mean 1.6-year follow-up [19]. Till et al. found that 75% of the cases showed stabilization or improvement in cognitive function at 15 months follow-up, but the improvement rate was less than that of healthy controls [21]. The variations in the results of the studies may be related to the differences in follow-up periods and evaluation methods used.

The reason for the improvements observed in children may be the ongoing development in the brain and neural plasticity. Brain development and neural plasticity may be involved in the success of cognitive rehabilitation and cognitive reserve enhancement approaches. Alternatively, cognitive performance in patients with pediatric MS may deteriorate in the long term relative to their baseline performance and the performance of their peers. This worsening cognitive state can be explained by impairment of the maturation and development of the immature central nervous system by demyelinating and neurodegenerative processes.

## 3. Social Cognition

Social cognition, a distinct area of cognitive function, refers to “the mental operations that underlie social interactions, including perceiving, interpreting, and generating responses to the intentions, dispositions, and behaviors of others” [23]. The theory of mind is an essential domain of social cognition, defined as the ability to infer other people's mental states, including beliefs, desires, knowledge, and intentions, hence explaining and predicting their behavior [24]. Social cognitive impairment has been studied in autism, psychiatric disorders, and developmental and neurodegenerative diseases. However, studies on social cognition in pediatric MS are very limited, with only one study published in the literature. Charvet and colleagues examined social cognition in 28 patients with pediatric-onset MS and 38 healthy controls. They used the SDMT and all three theories of mind tasks: reading the mind in the eyes test, the faux pas test, and the false-belief task. Patients with pediatric-onset MS performed worse than healthy controls on all theories of mind tasks, including both affective (eyes test) and cognitive (faux pas test and false-belief task) measures. Information processing speed, which was measured by SDMT, was slower in the pediatric-onset MS group (38%) than in the healthy control group (6%). However, when logistic regression analysis was performed to assess the influence of information processing speed on the theory of mind tasks, they suggested that information processing speed had no influence on the theory of mind [25]. Social cognitive impairment has been reported in adult patients with MS who were otherwise cognitively intact [26, 27]. Social cognition is important for the ability to form and maintain personal relationships. The effect of MS on social cognition is particularly important in patients with pediatric-onset MS since their social cognition skills are still undergoing development [25–27]. More studies on social cognition in pediatric MS are needed for the identification and prevention of social adjustment problems in these patients.

## 4. Neuropsychological Test Batteries for Pediatric MS

The aforementioned studies applied neuropsychological tests developed for adults to pediatric patients with MS. There is a need for a standardized, well-approved neuropsychological battery to detect MS-related deficits in pediatric patients. It is important to establish neuropsychological test batteries for evaluating cognitive domains that appear more frequently in children than adults and for assessing cognitive maturation in this age group. Several studies have attempted to establish such a neuropsychological test battery and to measure the sensitivity of presently available tests.

In an Italian multicenter study, Portaccio et al. validated a brief neuropsychological battery that they derived from the extensive battery used by Amato and colleagues. The researchers aimed to develop a Brief Neuropsychological Battery for Children (BNBC) with MS. The extensive neuropsychological battery involved the Wechsler Intelligence Scale for Children-Revised (WISC-R), the Selective Reminding Test (SRT, SRT-Delayed) from Rao's Brief Repeatable Battery (BRB), the Spatial Recall Test (SPART, SPART-D) from the BRB, the Symbol Digit Modalities Test (SDMT) from the BRB, Trail Making Test (TMT-A and TMT-B), Modified Card Sorting Test, Semantic Verbal Fluency Test (SVFT), Phonemic Verbal Fluency Test (PVFT), the Oral Denomination Test from the Aachener Aphasia Test, Token Test, the Indication of Pictures from the Neuropsychological Examination for Aphasia, and the Phrase Comprehension Test from the Battery for the Analysis of Aphasic Deficits (Table 3). Cognitive functions were assessed in 61 patients with childhood and juvenile MS and in 58 healthy controls. In this study, cognitive impairment was found in 41% of MS patients. Verbal and spatial memory (SRT and SPART), attention and concentration (SDMT and TMT), and language abilities (SVFT and Token Test) were the most frequently affected cognitive domains. Discriminant function analysis showed that the SDMT, the TMT-B, the Selective Reminding Test-Consistent Long-Term Retrieval (SRT-CLTR), and the vocabulary test from the WISC had higher discriminating ability. Therefore, using these tests, the researchers created the BNBC, which had a sensitivity of 96% and a specificity of 76% [28]. (Table 4).

Smerbeck et al. investigated the sensitivity and validity of two visual processing tests in 43 children with pediatric MS and 45 healthy controls: the Brief Visuospatial Memory Test-Revised (BVMT-R) and the SDMT. Previous large cross-sectional studies had demonstrated the sensitivity of these tests in adults. They found statistically significant differences between children with MS and healthy controls on BVMT-R Total Learning, BVMT-R Delayed Recall, and SDMT. These findings indicated that two relatively brief measures of visual-cognitive processing could be successfully applied to the pediatric population and could be useful in detecting and monitoring significant cognitive impairment [29].

Charvet et al. evaluated the SDMT as a screening tool for identifying pediatric onset MS patients at risk for cognitive impairment. The SDMT showed 77% sensitivity and 81% specificity for detecting neuropsychological impairment when administered within 1 year. Age and EDSS score were negatively correlated with SDMT score. The researchers concluded that these results support the use of the SDMT as a screening tool for cognitive function in pediatric MS [30].

## 5. Neuroradiologic Studies and Anatomic Correlation of Cognitive Impairment in Pediatric MS Patients

Several neuroradiologic studies have reported a correlation between deficits in executive function and atrophy of the thalamus and frontal lobes, as well as the correlation of a decrease in cognitive speed and mathematical performance with corpus callosum damage [31–33].

To normalize total and regional brain volumes for head size in their MRI correlations, Till and colleagues calculated cortical gray matter, thalamic, and global brain volumes using a scaling factor computed using the normalization of atrophy method. In addition, they also calculated T1- and T2-weighted lesion volumes in MS patients. Thirty-five pediatric-onset MS patients and 33 healthy controls were included in the study. Thalamic volume, corpus callosum area, normalized brain volume, and normalized gray matter volume were significantly lower in patients with MS than in healthy controls. Thalamic volume was strongly and positively correlated with an index global intellectual function in MS patients. MS patients with cognitive impairment did not differ from MS patients without cognitive impairment with regard to T1 or T2 lesion volume, normalized grey matter volume, or normalized brain volume. Neuropsychologic test performance was highly correlated with global and regional brain volume and less strongly correlated with T1 and T2 lesion volumes. The researchers suggested that cognitive impairment was related to the neurodegenerative component of MS [9].

The cerebellum is another anatomical area associated with cognitive function. It is a strategic node in various networks such as coordination and cognitive-behavioral loops [34]. Weier et al. examined associations between cognitive outcomes and cerebellar volume independent of cerebral volume in patients with pediatric MS. Twenty-eight pediatric-onset MS patients and 33 age- and sex-matched healthy controls were included in this study. All subjects underwent structural MRI and neuropsychological evaluation to assess intelligence, attention, information processing speed, language, visuomotor integration, and fine-motor dexterity. Neuropsychological battery included the Wechsler Abbreviated Scale of Intelligence (WASI), the TMT-B, the SDMT-oral version, Beery Visual Motor Integration, the vocabulary subtest of the WASI, and the Grooved Pegboard test. Cognitive performance in patients with MS was reduced relative to that in the healthy controls in the domains of attention, information processing speed, expressive language, visuomotor integration, and fine motor dexterity. Cerebellar volumes did not differ between MS group and healthy controls; however, cerebellar posterior lobe volume and infratentorial lesion volume accounted for extra variance in cognitive measures of information processing (SDMT) and vocabulary within MS patients in regression analysis. The researchers suggested that in addition to cerebral volume, the cerebellar volume was another correlate of cognitive function in children and adolescents with MS [35].

In a neuroradiologic study, Rocca and colleagues performed a structural and functional MRI examination to investigate the mechanisms responsible for the presence and severity of cognitive impairment in pediatric patients with MS. A total of 35 pediatric patients with MS and 16 sex- and age-matched healthy controls were included in the study. The researchers used voxel-based analysis with advanced structural MRI techniques to determine the patterns of regional involvement of white and gray matter according to patient's cognitive profile. They also used functional MRI to quantify the resting-state functional connectivity of the default mode network (DMN). The BNBC was used to assess participants' cognitive function, and subjects with abnormal performance in two tests were classified as cognitively impaired. Among the patients with MS, 45% were classified as cognitively impaired. Spatial and verbal memory abilities, language, attention, and concentration were the most significantly involved cognitive areas. The results showed that cognitively impaired patients with MS had a higher occurrence of T2 lesions and white and gray matter damage, including atrophy and diffusivity abnormalities in the posterior region of the parietal lobes close to midline (precuneus, posterior cingulum, and corpus callosum). Reduced resting-state functional connectivity in the precuneus was observed in cognitively impaired patients, whereas cognitively preserved patients showed increased resting-state functional connectivity in the anterior cingulate cortex. The researchers concluded that the presence and severity of cognitive impairment were associated with structural and functional abnormalities of the posterior core regions of the DMN in pediatric patients with MS [36].

In these neuroradiological studies, researchers used different parameters such as global brain volume, thalamic volume, gray matter volume, T1-T2 lesion volume, and cerebellar volume. Cognitive impairment has been related to T2 hyperintense lesions, diffuse white matter damage, and cortical and deep gray matter atrophy in adult MS patients [37]. Neuropathologic findings have revealed that MS affects regions that are functionally or anatomically involved in cognitive processes, such as cortical and deep gray matter, hippocampus, white matter, including normal appearing white matter in adult patients [38]. Consistent with the findings in adult patients, studies in pediatric MS patients suggest that a relationship exists between cognitive impairment and gray matter atrophy, white matter atrophy, and global and regional brain volume [9, 36].

## 6. Conclusions

A third of the pediatric MS patients experience cognitive impairment. In pediatric cases, the influence of demyelination on the developing central nervous system and neural network affects cognitive function negatively; on the other hand, neural plasticity and ability of compensation can have a positive effect on cognitive functions. Unlike in adults, intelligence and language functions are affected in pediatric MS. Young age at disease onset increases the risk of cognitive impairment. Although varying findings have been reported for the relationship between motor disability and cognitive disability, cognitive impairment may also occur independently from motor disability. Cognitive reserve is important both for the detection of patients with high risk and for the development of cognitive-protective approaches and rehabilitation. Knowledge about social cognition is limited in pediatric MS. Cognitive impairment in pediatric MS is a critical problem that has long-term effects on patients' academic and social quality of life; thus, the development of appropriate neuropsychological test batteries is important for screening patients who are at risk. Current studies have shown that SDMT and Brief Neuropsychological Battery for Children (BNBC) could be effectively used for screening cognitive functions in pediatric MS.

MRI studies have suggested that cognitive impairment in pediatric MS can be related to both gray matter atrophy and white matter atrophy and global and regional brain volume. More longitudinal studies with standardized neuropsychological batteries and advanced MRI techniques are needed to explore the mechanism of cognitive impairments and predict high-risk group. Understanding the pathogenesis of impaired cognition and the involvement of specific cognitive areas in pediatric MS will guide the development of treatment and rehabilitation, including cognitive rehabilitation, about which limited data are available in pediatric MS patients.

Assessment of cognitive function is also important for the evaluation of response to treatment. While defining the response to treatment and disease activity in pediatric MS, preservation of age-expected global and regional brain growth and age-expected cognitive maturation and function should be assessed, as well as the number of relapses, disability progression, and MR findings such as new, enlarging, or enhancing lesions.

## Figures and Tables

**Table 1:** Methods and results of cross-sectional studies.

Authors	MS sample size	Control sample size	Cognitive impairment definition	Neuropsychological tests	Impaired cognitive domains	Impairment rate
Banwell and Anderson [6]	10	—	Each individual's performance on each measure was scored relative to age norms.	Full-Scale IQ, Verbal IQ, Performance IQ, Verbal Comprehension Index, Perceptual Organization Index, Freedom from Distractibility Index, Processing Speed Index, CELF, COWAT, CELF formulated sentences, TLC Ambiguous sentences, VMI, Rey-O Figure, Grooved Pegboard, Vigilance subtest, CMS, CAVLT, WJRTA, WRAT	Language, visuomotor integration, verbal and visual memory, information processing speed, working memory and executive functions, general cognition	

MacAllister et al. [7]	37	—	Scores > 1.5 SDs below the published normative means on least 2 cognitive tasks	TMT, COWAT, Boston Naming Test, CELF-3rd edition, two subtests of the WRAML: Verbal Learning and Visual Learning, VMI	Complex attention receptive language, naming, memory	35%

Amato et al. [8]	63	57	Scoring less than the 5th percentile of healthy control performance on least 3 tests	WISC-R, SRT and SRT-D from the Rao's BRB, SPART and SPART-D from the BRB, SDMT from the BRB, TMT A and B, Modified Card Sorting Test, Semantic and Phonemic Verbal Fluency Test, and Oral Denomination Test from the Aachener Aphasia Test	Complex attention, visual and verbal memory, executive functions, language function	31%

Till et al. [9]	35	33	Three or more test scores 1.5 SDs below the normative values on the test battery	WASI, TMT-A and B, SDMT-Oral version, Visual Matching from the Woodcock–Johnson III (WJ-III) Test of Cognitive Abilities, Rapid Picture Naming from the WJ-III Test of Cognitive Abilities, Conner's Continuous Performance Test—5th edition, WSR, WMI-5th edition, Verbal Fluency subtest from the Delis–Kaplan Executive Function System, Picture Vocabulary from WJ-III Tests of Academic Achievement, Vocabulary and Similarities subtests from the WASI, WCST	Attention, information processing speed, expressive language, visuomotor integration	29.4%

Julian et al. [10]	187 (MS) 44 (CIS)	—	≥33% of test scores < 1 SD below normative data	WASI Full 2, Wechsler Individual Achievement Test II Pseudoword Decoding, Expressive One-Word Picture Vocabulary Test, Digit Span test, Wechsler Intelligence Scale for Children Coding Test, Contingency Naming Test, Delis Kaplan Executive Function System Trail Making Test, California Verbal Learning Test-Child version or II, WMI-6th edition, Wechsler Abbreviated Scale of Intelligence Matrix Reasoning, Grooved Pegboard Test	Fine motor speed, visuomotor integration, information processing speed	35% (MS) 18% (CIS)

BRB: Rao's Brief Repeatable Battery; CAVLT: children's auditory verbal learning test; CELF: clinical evaluation of language fundamentals; CMS: children's memory scale; COWAT: controlled oral word association test; Rey-O: Rey–Osterreith complex figure; SDMT: Symbol Digit Modalities Test; SRT: Selective Reminding Test; SRT-D: Selective Reminding Test-Delayed; SPART: Spatial Recall Test; SPART-D: Spatial Recall Test-Delayed; TMT: Trail Making Test; TLC: test of language competence; VMI: Beery–Buktenica visual motor integration test; WASI: Wechsler Abbreviated Scale of Intelligence; WCST: Wisconsin card sorting test; WISC-R: Wechsler Intelligence Scale for Children-Revised; WJ: Woodcock Johnson; WJRTA: Woodcock Johnson-revised tests of achievement; WRAML: wide range assessment of memory and learning test; WRAT: wide range achievement test; WSR: word selective reminding.

**Table 2:** Methods and results of longitudinal follow-up studies.

Authors	MS sample size at follow-up	Healthy controls sample size at follow-up	Duration of follow-up	Neuropsychological tests	Rate of decline in MS sample	Impaired cognitive domains
Amato et al. [16]	56	—	2 years	SRT, SRT-D, SPART, SPART-D, SDMT, TMT-A, TMT-B, Tower of London Test, Semantic and Phonemic Verbal Fluency Test, Oral Denomination Test from the Aachener Aphasia Test	70% (failure on at least 3 tests) (change in CCI between baseline and 2 years) Deteriorating: 75% Stable: 11.5% Improving: 13.5%	Verbal memory, complex attention, verbal fluency, receptive language

Amato et al. [17]	48	—	5 years	SRT, SRT-D, SPART, SPART-D, SDMT, TMT-A, TMT-B, Tower of London Test, Semantic and Phonemic Verbal Fluency Test, Oral Denomination Test from the Aachener Aphasia Test	38% (failure on at least 3 tests) (change in CCI between baseline and 5 years) Deteriorating: 56% Stable: 18.8% Improving: 25% (change in CCI between years 2 and 5) Deteriorating: 22.9% Stable: 10.4% Improving: 66.7%	Visual–spatial learning, expressive language

MacAllister et al. [18]	12	—	21.58 ± 9.3 months	TMT, COWAT, Boston Naming Test, CELF 3rd edition, WRAML, WMI	5/12 (cognitive decline assessed using the criteria of more tests showing impaired at the time of the 2nd test than at the baseline) 7/12 (cognitive decline assessed using the criteria of decline in *z*-score composite from the baseline to the time of the 2nd test)	Attention, executive functions, memory

Charvet et al. [19]	62 (MS) 5 (CIS)	—	1.64 ± 0.63 years	WASI, WASI-II, CVLT-C, CVLT-II, EOWPVT, WIAT-II, VMI, Digit Span subtest from WISC-IV, WAIS-IV, TMT	Rate of impairment was 37% at baseline and 33% at follow-up Most participants showed no change while *n* = 4 (6%) declined on two or more tasks and *n* = 6 (9%) improved on 2 or more tasks	Visuomotor integration, information processing speed, attention

Hosseini et al. [20]	35	—	Neuropsychological evaluations were conducted at baseline and up to four more assessments, with each evaluation separated by a minimum of 1 year.	SDMT, TMT, WASI, WISC-III	This study examined changes in cognitive maturation in a cohort of pediatric-onset MS patients as a function of age at disease onset and cognitive reserve factors (baseline IQ and parental social status) Younger age at disease onset was associated with a greater likelihood of cognitive decline on both the TMT-B (*p* = 0.001) and SDMT (*p* = 0.005)	Younger age at disease-onset increases the vulnerability for disrupted performance on measures of information processing, visual scanning, perceptual/motor speed, and working memory

Till et al. [21]	28	26	13–16 months (mean: 15 months)	WASI, Visual Matching from the WJ Tests of Cognitive Abilities, TMT A-B, SDMT, Conner's Continuous Performance Test (5th edition)–number of omission errors, Vocabulary and Similarities subtests of the WASI, WJ Tests of Achievement, Verbal Fluency Test from DKEFS, VMI, Block Design and Matrix Reasoning subtests from the WASI, TOMAL-2, WSR, Grooved Pegboard test	Improving: 18% in MS group, 86% in control group The RCI method results showed that the majority of patients remained stable or improved over time (21 of 28; 75%), with only seven of 28 patients (25%) demonstrating decline on three or more of the neuropsychological tests. Only 1 of 26 controls (3.8%) showed cognitive deterioration (*p* < 0.05).	Attention, information processing speed, visuomotor integration, verbal fluency, visual memory, calculation, spelling ability

CCI: cognitive change index; CVLT: California Verbal Learning Test; DKEFS: Delis–Kaplan executive function system; EOWPVT: Expressive One-Word Picture Vocabulary Test; RCI: reliable change index; TOMAL-2: test of memory and learning-2nd edition; WAIS-IV: Wechsler adult intelligence scale 4th edition; WIAT-II: Wechsler Individual Achievement Test-2nd edition.

**Table 3:** Neuropsychological tests.

Neuropsychological test	Cognitive domain
WISC-R (Wechsler Intelligence Scale for Children)	IQ (intelligence quotient)

SRT-LTS (Selective Reminding Test-Long-Term Storage) SRT-CLTR (Selective Reminding Test-Consistent Long-Term Retrieval) SRT-D (Selective Reminding Test-Delayed) SPART (Spatial Recall Test) SPART-D (Spatial Recall Test-Delayed)	Memory

MCST (Modified Card Sorting Test)	Abstract/conceptual reasoning

SDMT (Symbol Digit Modalities Test) TMT-A/ B (Trail Making Test A/B)	Attention/concentration

SVFT (Semantic Verbal Fluency Test) PVFT (Phonemic Verbal Fluency Test) IPT (Indication of Pictures Test) PCT (Phrase Comprehension Test) Token Test ODT (Oral Denomination Test)	Language

**Table 4:** Brief Neuropsychological Battery for Children (BNBC).

(i) SRT
(ii) SDMT
(iii) TMT
(iv) Vocabulary test from the WISC-R

Duration: 30 minutes. Sensitivity (96%), specificity (76%).
